# Profound vitamin D deficiency in four siblings with Imerslund‐Grasbeck syndrome with homozygous CUBN mutation

**DOI:** 10.1002/jmd2.12072

**Published:** 2019-07-26

**Authors:** Jose I. R. Ciancio, Mark Furman, Siddharth Banka, Stephanie Grunewald

**Affiliations:** ^1^ Metabolic Medicine Department Great Ormond Street Hospital for Children NHS Foundation Trust London UK; ^2^ Consultant Paediatric Gastroenterologist Royal Free Hospital London UK; ^3^ Division of Evolution and Genomic Sciences, School of Biological Sciences, Faculty of Biology, Medicine and Health The University of Manchester Manchester UK; ^4^ Manchester Centre for Genomic Medicine, St. Mary's Hospital Manchester University, NHS Foundation Trust, Health Innovation Manchester Manchester UK

**Keywords:** Cubilin, Imerslund‐Gräsbeck syndrome, tubular proteinuria, vitamin B12 malabsorption, vitamin D deficiency

## Abstract

Imerslund‐Grasbeck syndrome (IGS, OMIM 261100) is a rare autosomal recessive disease characterized by vitamin B12 malabsorption resulting in megaloblastic anemia and asymptomatic proteinuria. IGS is caused by bi‐allelic mutations in either CUBN or AMN that respectively encode the cubilin and amnionless subunits of the cobalamin‐intrinsic factor receptor. We report four siblings (three boys, one girl) of non‐consanguineous parents of Jewish background, aged 10 months to 12 years, with homozygous CUBN frameshift c.2614_2615deIGA p.(Asp872LeufisTer3) mutation and typical features of IGS. The two older brothers presented in early infancy with lethargy, mouth ulcerations, eosinophilic enterocolitis, megaloblastic anemia and failure to thrive. Investigations revealed low serum cobalamin levels. Intramuscular hydroxycobalamin supplementation resulted in dramatic resolution of all symptoms including lethargy. A positive impact on their growth curve was seen. Prospective early treatment in the younger siblings prevented these manifestations. Proteinuria with proximal tubulopathy was seen in all patients, plasma protein level and renal function were normal. All children had pronounced vitamin D deficiency and required high doses of oral supplementation. Vitamin B12 treatment could be individually adjusted; requirement decreased with age. Tubulopathy showed improvement over time. Low vitamin D could be explained by cubilin being involved in reabsorption of vitamin carriers.

## 1 BACKGROUND

Imerslund‐Grasbeck syndrome (IGS, OMIM 261100) is a rare autosomal recessive disease characterized by selective intestinal vitamin B12 malabsorption and proteinuria with unaffected renal function. Untreated, it results in megaloblastic anemia, failure to thrive, recurrent infections and neurological damage.^1^, ^2^ It was first described in the 1960's in Finland and Norway and over the years, it has been described in other populations (Turkish, Israeli, and Saudi Arabia).^3^, ^4^

IGS is caused by bi‐allelic mutations in either the gene encoding for cubilin (CUBN; 602 997) or amnionless (AMN; 605 799), subunits of the cobalamin‐intrinsic factor complex receptor (Cubam). Cubilin functions as a multi‐ligand receptor by binding to the transmembrane protein Amnionless.

The Cubam receptor is highly expressed in the small intestine and also in the proximal tubules of the kidneys where it is involved in the reabsorption of low molecular weight plasma proteins including vitamin carriers and lipoproteins.^5^

Vitamin B12 is an essential cofactor for methionine synthase, which catalyzes the conversion of homocysteine to methionine in cytoplasm; and mitochondrial methylmalonyl‐CoA mutase, which catalyzes the conversion of methylmalonyl‐CoA to succinyl‐CoA. A deficiency of vitamin B12 results in elevated methylmalonic acid (MMA) and homocysteine; and decreased levels of methionine in serum and urine. It also has an important role in cellular metabolism, especially in red cell DNA synthesis leading to immature and dysfunctional red cells (megaloblasts) and mitochondrial metabolism.^5^, ^6^ Clinical B12 deficiency with classic haematological and neurological manifestations is relatively uncommon. However, subclinical deficiency affects between 2.5% and 26% of the general population.^7^

The most common causes of B12 deficiency in adults include: autoimmune pernicious anemia (PA); dietary deficiency (general malnutrition, vegetarian or vegan diet, and chronic alcoholism) or intestinal malabsorption secondary to other disorders (ie, coeliac disease, ileal resection, bacterial overgrowth); gastric disease or surgery (partial or complete gastrectomy or gastric reduction surgery causing deficiency of intrinsic factor) and pancreatic disease or pancreatectomy (B12 is not released from haptocorrin due to insufficient pancreatic enzyme activity).^7^, ^8^ In newborns B12 deficiency can occur due to maternal PA or in exclusively breast‐fed children of mothers with B12 deficiency.^9^ Genetic causes include transcobalamin deficiency, intrinsic factor deficiency (IFD) and defects in the Cubam receptor.^3^, ^4^, ^5^, ^6^, ^10^ Furthermore, genetic predisposition to PA is also part of common organ‐specific autoimmune diseases.^11^

The clinical picture and laboratory abnormalities show a significant overlap in IGS and IFD and are characterized by megaloblastic anemia and low vitamin B12. The presence of accompanying asymptomatic proteinuria is specific for IGS. In the past, the Schilling Test was used to differentiate both conditions. Currently genetic testing is used as first line of investigation^.^ 1, 12, 13 Tanner et al propose a genetic diagnostic strategy for intestinal malabsorption of cobalamin based on the ethnicity of the patient, giving first priority to the search of founder mutations, followed by other known mutations as second priority and finally screening remaining exons.^10^

## 2 CASE REPORT

We report on four siblings (three boys, one girl), aged between twelve years and eight months old (patients A to D). The family is of Jewish Ashkenazi background with no consanguinity.

Patient A was born at term after an uncomplicated pregnancy, no family history of relevance and no history of maternal dietary restrictions. He was breast‐fed exclusively for five weeks and subsequently commenced on formula feed top‐ups. He presented during the first year of life with lethargy, mostly described as an “unhappy” baby with no energy. He suffered from recurrent mouth ulcerations, recurrent diarrhea and failure to thrive (weight and height on 2nd centile) which prompted investigations for cow's milk protein allergy and coeliac disease that showed normal results. These tests revealed the presence of megaloblastic anemia and low vitamin B12 level, for which he was started on intramuscular hydroxocobalamin (initially every six months) and referred to our department at four years of age.

Subsequent extended metabolic investigations showed decreased total homocysteine of 1 μmol/L (5‐15), normal plasma methionine 14 μmol/L (10‐60); serum folate 7.2 μg/L (3.0‐20.0) and slightly increased plasma MMA of 0.5 μmol/L (0‐0.28). Urine organic acids and qualitative urine aminoacid pattern were normal. It is important to note that these tests were taken shortly after a dose of intramuscular hydroxocobalamin was given. The ratio of N‐Acetyl‐β‐D‐glucosaminidase (NAG)/Creatinine, a marker for proximal tubular function, was elevated at 152 Units/mmol (normal range: 2‐22), accompanied by normal plasma albumin level and normal renal function. The laboratory findings and clinical symptoms showed dramatic improvement from the very first dose of vitamin B12 including complete resolution of mouth ulcerations, increased energy levels and positive impact on his growth curve.

Autozygosity mapping was performed as previously described^14^ revealing multiple areas of homozygosity including a 4.6 Mb region on chromosome 10 that encompassed *CUBN*. Sanger sequencing of *CUBN* revealed a frameshift c.2614_2615deIGA (p.(Asp872LeufisTer3)) (NM_001081.3) variant that was homozygous in patient A and heterozygous in both parents. This mutation has been previously reported in homozygous state in two patients with Jewish Ashkenazi background.^3^ Hence, the diagnosis of Imerslund‐Grasbeck syndrome was confirmed.

Patient B had early failure to thrive on a background of frequent vomiting. Similar to his older brother he was quite an irritable child. At 12 months of age he was found to be vitamin B12 deficient (48 ng/L) and on urine testing he was also found to have proteinuria and an increased NAG/Creatinine excretion (135 U/mmol).

Subsequent targeted investigation for the familial mutation confirmed the diagnosis in patient B and also revealed two younger siblings to also being affected (Patients C and D). The latter two were prospectively treated with IM hydroxocobalamin from birth and remain asymptomatic except for abnormal NAG/Creatinine ratio and low vitamin D.

Hydroxocobalamin was given on a weekly basis at the beginning of the treatment in patients A and B. The doses were subsequently spaced depending on the serum Vitamin B12 results in each sibling aiming for levels >250 ng/L. Currently it is given every three to four months in all four patients.

**Figure 1:**
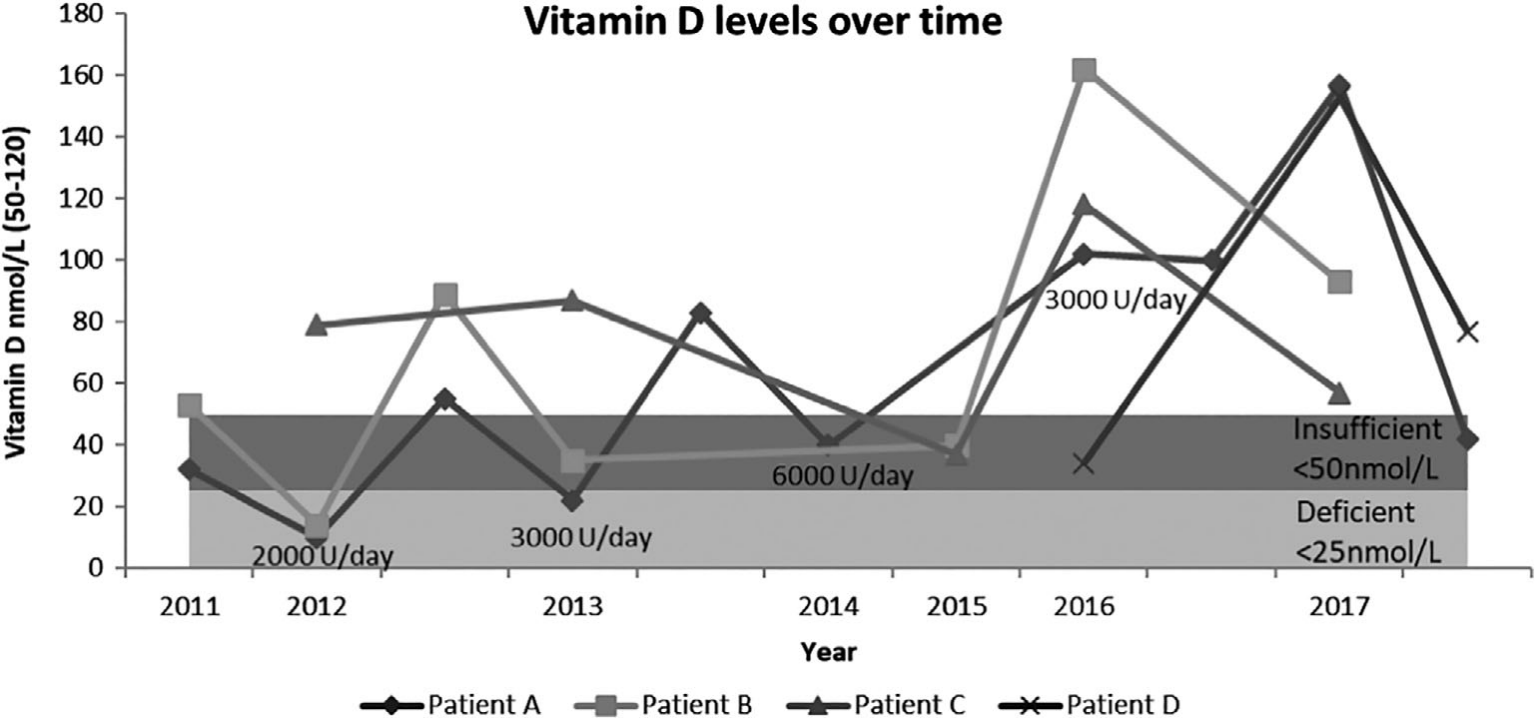
Level of plasma 25 (OH) vitamin D over time. Deficient level (<25 nmol/L). Insufficient level (<50 nmol/L). Patients A and B showed persistently insufficient levels (<50 nmol/L) despite oral supplementation in high doses (U/day)

Another interesting aspect that all patients share is persistent vitamin D insufficiency (Figure 1). In order to maintain levels above the insufficient range patients A and B required high doses of oral supplementation (up to 6000 U/day). Patients C and D achieved normal levels on 3000 U/day dramatically dropping when supplementation is stopped.

## 3 MATERIALS AND METHODS

Total 25‐hydroxyvitamin D (D2 + D3) was measured using a chromatography‐mass spectrometry method on a Waters Xevo TQ‐S tandem mass spectrometer. Normal range in children: 50 ‐ 120 nmol/L; Insufficient: 25‐50 nmol/L; Deficient: <25 nmol/L.

Vitamin B12 was measured using a solid‐phase competitive chemiluminescent enzyme immunoassay on a Siemens Immulite 2500. Normal range: 183‐1090 ng/L.

Plasma MMA was determined by Liquid Chromatography‐Tandem Mass Spectrometry (LC‐MS/MS). Normal range: 0‐0.28umol/L.

Plasma amino acids were measured by HPLC (reverse phase high performance liquid chromatography) on a Waters Alliance 2695 HPLC analyzer and total homocysteine was measured by ion exchange chromatography in plasma (on a Biochrom 30+ amino acid analyzer with dithiothreitol as reducing agent).

Urine organic acids were measured by chromatography mass spectrometer method on an Agilent 7890A GC‐MS analyzer.

N‐Acetyl‐β‐D‐glucosaminidase (NAG)/Creatinine ratio was determined by colorimetric NAG assay kit (Roche Diagnostics) on a Werfen IL650 clinical chemistry analyzer.

Routine urinalysis was performed using Clinitek Multistix urine test strips.

Qualitative aminoacid pattern was measured by thin layer chromatography.

Autozygosity mapping was performed with Genome‐Wide Human SNP Array 6.0. Genotype calls were generated with the use of the Birdseed V2 algorithm within the same software, and the results were analyzed by AutoSNPa.

## 4 DISCUSSION

Detection of *CUBN* (c.2614_2615deIGA p.(Asp872LeufisTer3) mutation in our patients further confirms this variant to be a founder mutation in Ashkenazi Jewish population.

All four children presented with evidence of proximal tubular damage that seems to improve with age (Table 1) although it continues to be abnormal at present. Proteinuria has been described in many IGS cases though its absence does not exclude the diagnosis. The four siblings in this case report seem to be showing a comparable clinical progress. Other cases in literature describe how patients that share a mutation can present with different degree of severity and progression. Almadani et al. described a case of two siblings where only one of them presented proteinuria as a late symptom with worsening renal function over the years.^15^

**Table 1:** Initial and current NAG/creatinine levels

Patient	Sex	Date of birth	Initial urine	Most recent urine
			NAG/creat ratio	NAG/creat ratio
A	M	04/09/2004	152 U/mmol	119 U/mmol
			(4 years old)	(12 years old)
B	M	01/06/2007	135 U/mmol	80 U/mmol
			(5 years old)	(9 years old)
C	M	10/11/2011	200 U/mmol	40 U/mmol
			(10 months old)	(5 years old)
D	F	27/07/2016	622 U/mmol	
			(8 months old)	
			Normal 2‐20 U/mmol	

Abbreviation: NAG, N‐Acetyl‐β‐D‐glucosaminidase.

Storm et al described a correlation between mutation type and presence or absence of the characteristic low molecular weight proteinuria in nine IGS patients. Certain mutations affect only receptor recognition of IF‐B12 in the small intestine and others affect the overall expression of cubilin on both intestinal and proximal tubules cell surfaces.^4^

Cubilin interacts with the endocytic receptor megalin to allow the reabsorption of plasma proteins in the kidneys. These include vitamin D binding protein (DBP).^16^ Nykjaer et al showed increased urinary excretion of steroid 25‐hydroxyvitamin D3 and vitamin D‐binding protein (DBP) in patients with a homozygous null CUBN mutation that result in premature stop mutation. Such a mutation is expected to result in complete absence of the CUBN protein due to nonsense‐mediated decay of the mutant transcript. Importantly, patients with missense mutations did not show any alteration in 25‐hydroxyvitamin D3 and DBP excretion. Hence, increased loss of 25‐hydroxyvitamin D3 and DBP seems to likely be a feature of bi‐allelic null mutations. Notably, our patients also have a frameshift variant and the mutant transcript is likely to undergo nonsense‐mediated decay. Our observation of vitamin D deficiency in affected patients is, therefore, consistent with the hypothesis proposed by Nykjaer et al.

No other potential secondary causes of hypovitaminosis D were identified in this group. The profound deficiency in this case series could be mutation specific supporting the concepts of phenotypic heterogeneity of this disease and mutation/proteinuria correlation.^4^

## 5 CONCLUSION

Imerslund‐Grasbeck syndrome should be suspected when there is low serum vitamin B12, especially when it is accompanied by megaloblastic anemia and proteinuria.

Intramuscular hydroxocobalamin treatment is effective in reversing the clinical and biochemical findings and preventing the manifestation of the disease in asymptomatic patients. The frequency of the doses should be adjusted individually according to clinical response and monitored by serum levels of vitamin B12 and ideally also measuring MMA, the most specific and sensitive single marker for B12 deficiency, often used as the “gold standard” for defining B12 status.^7^ Requirement was higher in the symptomatic siblings (subjects A and B) when compared to the prospectively treated siblings at the same age (subjects C and D).

The profound vitamin D insufficiency can be explained by the loss of vitamin carriers in urine and can potentially be a mutation specific characteristic. This was the only clinical symptom related to proteinuria we noticed in this group of patients. The loss of other low molecular proteins does not seem to cause relevant disturbances. We, therefore, recommend regular 25‐hydroxyvitamin D3 measurement and individually adjusted treatment.

NAG/Creatinine ratio improved with age although it has not completely normalized. Considering the deteriorating renal function seen in other published cases, we highlight the need for regular renal function monitoring for the follow‐up of IGS patients.^15^

## COMPLIANCE WITH ETHICS GUIDELINES

**Main author / guarantor: Jose Ignacio Rodriguez Ciancio**, planning, conduct, and reporting of the work described in the article.

**All the Co‐Authors were involved in the care of these patients and contributed with detailed information on diagnosis, treatment and follow‐up**.

**Mark Furman**, contributed with details of initial clinical presentation, diagnosis and laboratory results. Critical revision for intellectual content.

**Siddharth Banka**, contributed with details on molecular diagnosis, genotype and phenotype of the presentation. Critical revision for intellectual content.

**Stephanie Grunewald**, contributed with details on diagnosis and clinical progress of the patients. Critical revision for intellectual content.

**Jose Rodriguez Ciancio**, Mark Furman, Siddharth Banka and Stephanie Grunewald declare that they have no conflict of interest.
